# Mechanisms of mitochondrial quality control in autosomal recessive Parkinson’s disease

**DOI:** 10.1186/2193-1801-4-S1-L36

**Published:** 2015-06-12

**Authors:** Olga Corti

**Affiliations:** Institut du Cerveau et de la Moelle épinière, Paris, France

**Keywords:** Parkinson’s disease, mitophagy, ER/mitochondria interface

The genes encoding the E3 ubiquitin protein ligase Parkin (PARK2) and the mitochondrial serine/threonine kinase PINK1 (PARK6) are mutated in clinically similar, autosomal recessive early onset Parkinson’s disease (PD) forms. Over the past ten years, a number of studies in different model systems have demonstrated that PINK1 and Parkin regulate jointly several processes relevant to maintenance of mitochondrial quality, including mitochondrial trafficking and dynamics, mitophagy and biogenesis. By using a combination of approaches of cell biology, confocal imaging and biochemistry in different cell models, we recently showed that loss of protein import efficiency triggers recruitment of Parkin by PINK1 in proximity of the translocase of outer mitochondrial membrane (TOM). We provided evidence that the degradation of specific TOM subunits plays a key role in initiating the autophagic degradation of damaged mitochondria. We also showed that PINK1 and Parkin interact with the TOM machinery on polarized mitochondria. Our results suggests that this interaction modulates the import of the multifunctional mitochondrion-protective matrix enzyme 17beta-hydroxysteroid dehydrogenase 10, which is depleted in Parkin-deficient mice and Parkinson’s disease patients. Electron and confocal microscopy, and calcium imaging approaches used to characterize the endoplasmic reticulum (ER)-mitochondria interface, a compartment previously linked to neurodegenerative processes, revealed enhanced juxtaposition between these organelles, associated with increased ER-to-mitochondria calcium transfer in cells from Parkin-deficient mice and patients with PARK2 mutations. Our current work aims at investigating the relevance of mitochondrial quality control mechanisms regulated by PINK1 and Parkin in different cell types of the central nervous system, to evaluate the contribution of each of them to the physiopathology of autosomal recessive Parkinson’s disease.

